# Evidence from a breast cancer hypofractionated schedule: late skin toxicity assessed by ultrasound

**DOI:** 10.1186/1756-9966-32-80

**Published:** 2013-10-24

**Authors:** Valeria Landoni, Carolina Giordano, Annelisa Marsella, Biancamaria Saracino, Maria Grazia Petrongari, Anna Maria Ferraro, Lidia Strigari, Paola Pinnarò

**Affiliations:** 1 Laboratory of Medical Physics and Expert Systems, Regina Elena National Cancer Institute, Rome, Italy; 2 Department of Radiation Oncology Regina Elena National Cancer Institute, Rome, Italy; 3 Department of Radiology Regina Elena National Cancer Institute, Rome, Italy

**Keywords:** Breast cancer ultrasound tissue characterization, Radiation toxicity, Hypofractionactionated radiotherapy

## Abstract

**Background:**

Feasibility of whole breast hypofractionated radiotherapy schedules in breast conserving therapy is recognized however concerns remain about the role of the boost dose on the overall treatment’s potential toxicity. In this study we report on the possibility to quantitatively evaluate radiation induced toxicity in patients treated with an abbreviated course with major concern in the irradiated boost region.

**Methods:**

Eighty-nine patients who underwent conservative surgery for early-stage breast cancer followed by adjuvant accelerated hypofractionated whole breast radiotherapy were included in this study to assess skin and subcutaneous tissue late toxicity by means of ultrasonographic quantitative examination. For each patient the skin thickness was measured at four positions: on the irradiated breast, in the boost region and in the corresponding positions in the contra-lateral not treated breast. All patients were scanned by the same radiologist to reduce potential inter-operator variability, the operator was blind to the scoring of the patient CTCv3 late toxicity as well as patient treatment characteristics. Ultrasound assessment and clinical evaluation were compared.

**Results:**

The median time between the end of adjuvant radiotherapy and ultrasound examination was 20.5 months. The measured mean skin thickness in the irradiated breast was 2.13 ± 0.72 mm while in the mirror region of the contra-lateral healthy breast was 1.61 ± 0.29 mm. The measured mean skin thickness in the irradiated boost region was 2.25 ± 0.79 mm versus 1.63 ± 0.33 mm in the corresponding region of contra-lateral healthy breast. The mean increment in skin thickness respect to the counterpart in the healthy breast was 0.52 ± 0.67 mm and 0.62 ± 0.74 mm for the breast and the boost region respectively. A significant direct correlation was found between the increment in skin thickness in the irradiated breast and in the boost region with fibrosis (G ≥ 1).

**Conclusions:**

In this study results from a breast cancer hypofractionated schedule in terms of late skin toxicity are reported. In particular our study confirms that late cutaneous reactions can be reliably assed by ultrasonographic examination, also discriminating between regions irradiated at different doses, and that this instrumental evaluation is in agreement with clinical stated toxicity.

## Background

Several phase III randomized clinical trials [1-3] have evaluated the issue of hypofractionation in breast cancer showing that hypofractionated adjuvant whole breast radiotherapy (WBRT) after breast-conserving surgery offers disease control rates and toxicity profiles equivalent to those seen with normofractionated approach. Based on long-term results from these studies there is, therefore, a mature body of data supporting, as level I evidence, selected whole breast hypofractionated radiotherapy schedules in breast conserving therapy (BCT). However concerns remain about the role of the boost dose in hypofractionated fashion on the overall treatment’s potential toxicity.

In the aforementioned randomized trials, in fact, none [1] or only about 50% [2,3] of the patients received a boost dose to the tumor bed but always with a normofractionated approach (i.e., at 2 Gy/fr to a total dose of 10 Gy in five fractions). More recently several Authors [4-7] reported on accelerated schedules of WBRT with concomitant boost in prospective or retrospective studies. In October 2004 we began a phase II prospective clinical trial using an accelerated hypofractionated radiotherapy schedule consisting of 10 daily fractions of 3.4 Gy to whole breast plus a boost dose of 8 Gy in a single fraction in patients who underwent breast conserving surgery for early-stage breast cancer and who refused adjuvant conventional radiotherapy regimen (50 Gy in 25 daily fractions to the whole breast followed by 10–16 Gy in 5–8 daily fractions to the tumour bed) [4]. To quantitatively evaluate skin radiation induced late toxicity after an abbreviated course, with major concern in the irradiated boost region, patients underwent an ultrasonographic examination. In this article we report late normal-tissue toxicity assessment by a quantitative ultrasound technique and its relationship with clinical evaluation in the affected breast, as well the comparison with the contra-lateral healthy not irradiated one, after a minimum follow-up of 11.4 months. The analysis was performed in a cohort of patients who, between October 2004 and December 2010, adhered to the above-mentioned study.

## Methods

### Patients

Eighty-nine out of 152 patients who underwent conservative surgery for early-stage breast cancer (pTis, pT1-2, pN0-1) and who adhered, between October 2004 and December 2010, to our adjuvant accelerated hypofractionated whole breast radiotherapy prospective clinical trial were included in this study to assess skin and subcutaneous tissue late toxicity by means of quantitative ultrasonographic examination. The radiotherapy schedule consisted of 34 Gy in 10 daily fractions over 2 weeks to the whole breast, followed by an electron boost dose of 8 Gy in a single fraction to the tumour bed. Exclusion criteria included, pathologic diameter of primary > 3 cm, the need for radiotherapy to regional lymph nodes, prior breast or thoracic radiotherapy for any condition, synchronous or metacronous bilateral invasive or non-invasive breast cancer, age less than 18 years. The protocol has been approved by the local Ethics and Scientific Committee. All patients provided a written informed consent. Out of 89 patients, 36 (40%) were treated with adjuvant chemotherapy before radiotherapy, either with CMF (cyclophosphamide 600 mg/m2, methotrexate 40 mg/m2, 5-FU 600 mg/m2 d 1 and d8 q 4 weeks × 6) in 7 patients or FEC ( 5-FU 600 mg/m2, epirubicin 60 mg/m2, cyclophosphamide 600 mg/m2 d 1 q 3 weeks × 6) in 12 patients or EC (epirubicin 60 mg/m2, cyclophosphamide 600 mg/m2 d1 q 3 weeks × 4) followed by Docetaxel 100 mg/m2 d1 q 3 weeks × 4) in 17 patients. The adjuvant chemotherapy had generally been completed 3 to 4 weeks before starting radiotherapy. Adjuvant hormonotherapy, with tamoxifen (associated with luteinizing hormone-releasing hormone analogue in 1 patient) or anastrozole, or letrozole, if indicated, was given simultaneously with radiotherapy.

### Radiation therapy

Details of radiotherapy treatment and the radiobiological considerations were fully described in a previous paper [8]. Briefly 3D conformal radiotherapy was delivered by two opposed 6MV photon beams. Wedge compensation was used to ensure a uniform dose distribution to the target volume of -5% and +7% [9]. No bolus was positioned on the patient skin. The total dose was 34 Gy delivered in 10 daily fractions, 3.4 Gy per day, 5 days a week; the dose was normalized at the ICRU (International Commission on Radiation Units and Measurements) reference point [9]. The boost dose of 8 Gy (prescribed to the 90% reference isodose) was administered, after one week in a single fraction with electrons. Electron beam energy (range 6 to 12 MeV) was chosen according to tumour bed depth and thickness indentified by metallic clips purposefully positioned at the surgery time and/or by computer tomography images. Our schedule of 34 Gy in 10 fractions plus a boost of 8 Gy in one fraction is biologically equivalent (in respect of 2 Gy/fr conventional radiotherapy approach) to 47–53 Gy for whole breast and 59–70 Gy considering the tumour boost volume, according to an α/β range values from 4.6 to 10 Gy.

### Clinical toxicity assessment

Scale used to score toxicity was the National Cancer Institute Common Toxicity Criteria for Adverse Events version 3.0 (CTVv3) for skin and subcutaneous induration/fibrosis [10]. Effects of radiation therapy on skin and subcutaneous tissue were graded on 0 to 3 with G0 indicating no toxic effects, G1 = increased density on palpation, G2 = marked increase in density and firmness on palpation with or without minimal retraction, G3 = very marked density, retraction or fixation. Clinical toxicity assessment was performed the same day of instrumental exam by a radiation oncologist not involved in the ultrasonographic session.

### Ultrasonographic examination

**Figure 1:**
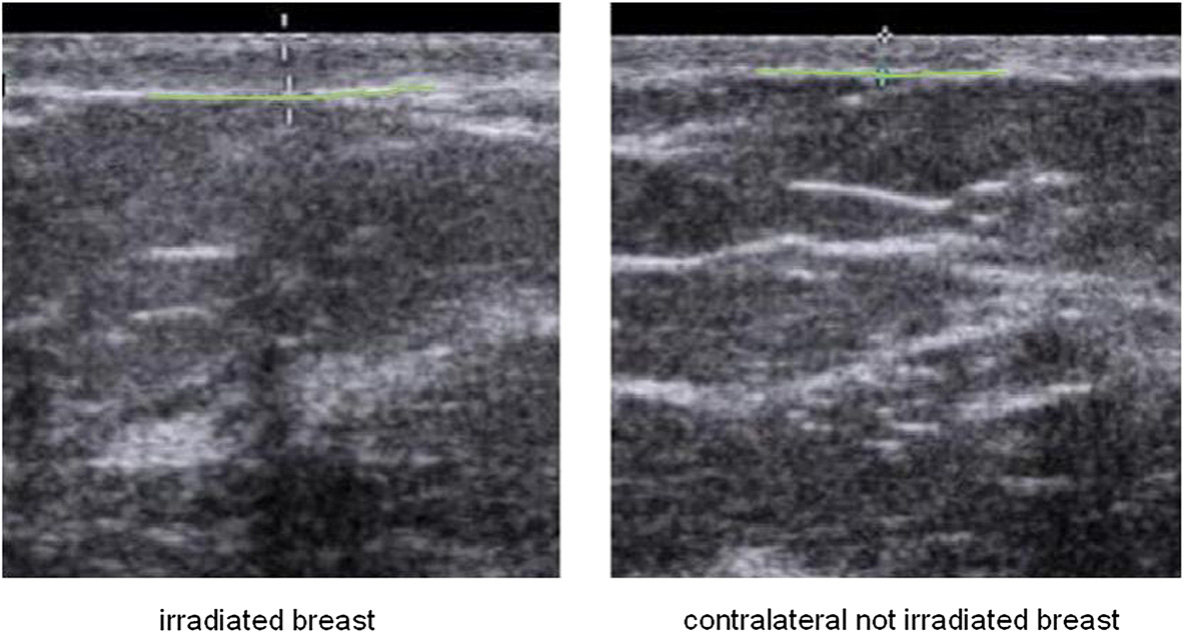
The full thickness, epidermis plus dermis was measured on the irradiated breast, in the boost region and in the corresponding positions in the contra-lateral not treated breast.

**Figure 2:**
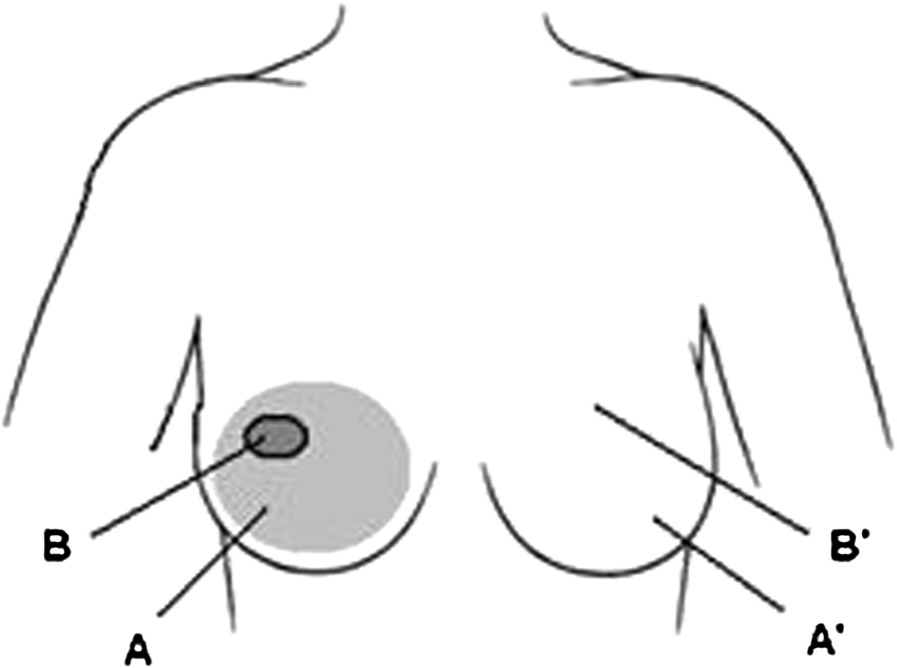
**Diagram of the location of the ultrasound measurements. A** corresponds to the irradiated breast at 34 Gy, **B** corresponds to the boost region at 42 Gy, **A’** and **B’** correspond to the mirror positions in the contra-lateral healthy breast.

Patients laid in supine position. A thin layer of ultrasound transmission gel was used to ensure good coupling between the skin and the probe. The axis of the transducer was kept perpendicular to the surface of the skin and the slightest possible force was applied to avoid affecting the skin thickness measurement. Four to six ultrasound scans were obtained for each region (radial and vertical). The boost region was identified from a picture of the radiotherapy field taken at the time of treatment. The ecographic exam took approximately 10–15 minutes. Images were acquired in B-mode using a Sequoia 512 scanner (Siemens Medical Systems, USA) with a linear transducer array transducer (15 L8 W). Frequency: 8.0 - 15.0 MHz. A “Breast protocol” was applied for imaging with the following characteristics: dynamic range was 68 dB, that allows optimal differentiation between subtle changes in echo intensities in the skin region, overall gain was set to 18 dB, Delta was Δ2 for high contrast resolution, focal zone was always placed so to optimize lateral resolution at the level of the skin. The full thickness, epidermis plus dermis was measured (Figure 1). Measurements were performed at four positions for each patient: on the irradiated breast at 34 Gy (A), on the irradiated breast in the boost region at 42 Gy (34 Gy whole breast + 8 Gy boost) (B), and in the corresponding positions in the contra-lateral not treated healthy breast (A’) and (B’). See Figure 2. All images were stored on disk for further analysis. All patients were scanned by the same radiologist to reduce potential inter-operator variability, the operator was blind to the scoring of the patient CTCv3 late toxicity as well as patient treatment characteristics.

### Statistical analysis

A t-test for independent samples was used to evaluate the correlation between skin thickness in the irradiated region and in the same region of the contralateral breast (A vs A’), the same was performed between skin thickness in the boost region and in the same region of the contralateral breast (B vs B’). Also a t-test for paired samples was used to evaluate the correlation between skin thickness in the boost region and in the non boost region in the irradiated breast (B vs A). To investigate the correlation between skin thickness and clinical and dosimetric variables measured the Pearson correlation coefficient and the Spearman correlation coefficient were calculated for continuous and ordinal variables respectively. A t test was then performed to state the significance of the correlation. For all the analysis the correlation was considered significant if p < 0.05.

## Results

Patient and tumour main characteristics are shown in Table 1.

**Table 1:** Patients and tumour characteristics

**Age (years)**	Median 62 (31–79)
**Menopausal status pre/post**	25/64
**pT stage**	
pTis	12
pT1	66
pT2 (≤3 cm)	11
**pN stage**	
pN0	70
pN1 (≤ 3 positive nodes)	19
**Estrogen receptor status**	
Positive/negative	76/13
**Progesteron receptor status**	
Positive/negative	76/13
**Chemotherapy yes/no**	36/53
**Hormonotherapy**	
No	20
Tamoxifen	35
Anastrozole	18
Letrozole	16
**Follow-up (months)**	20.5 (11.4-85.7)

**Figure 3:**
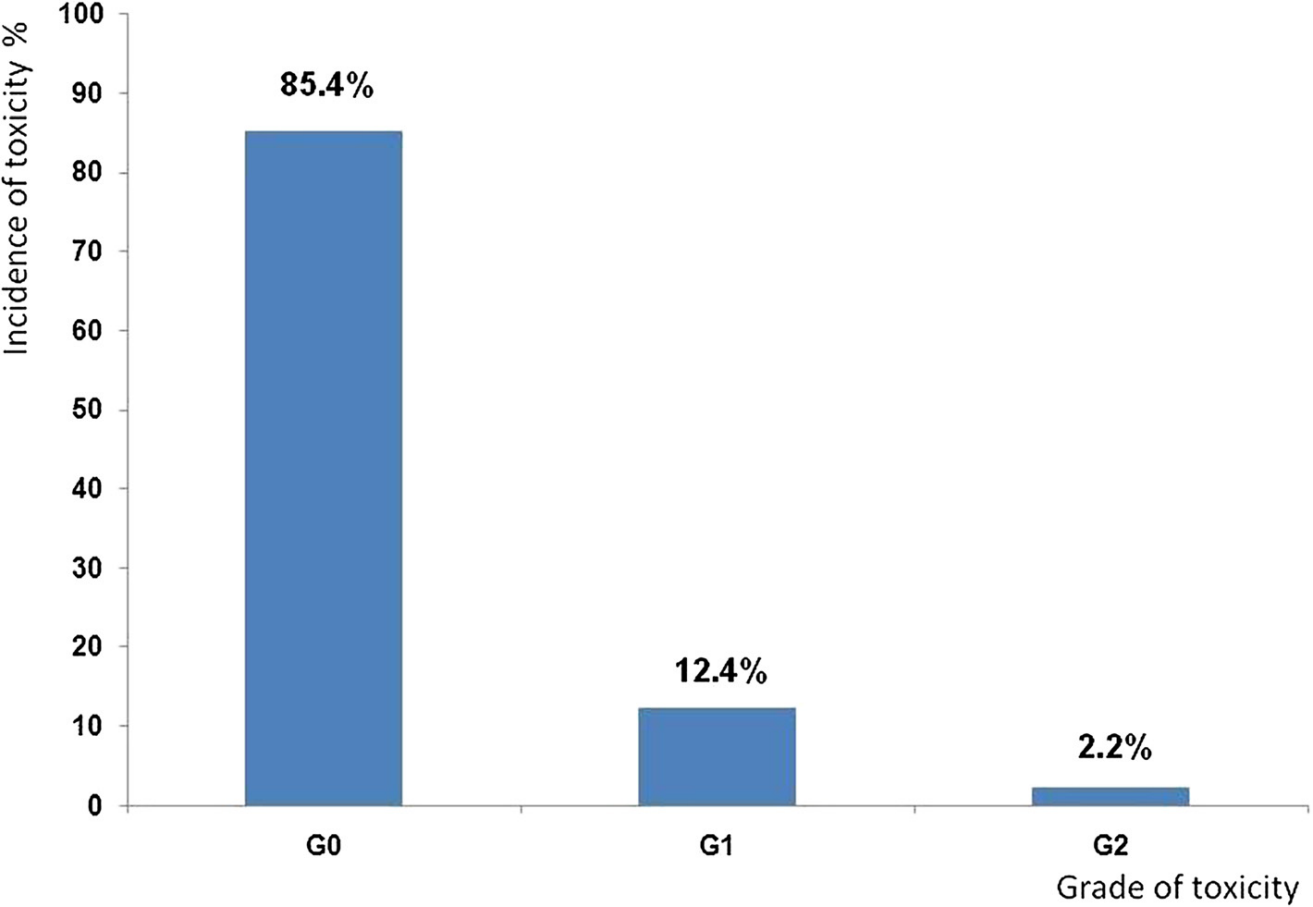
Percentage incidence of late skin toxicity.

**Figure 4:**
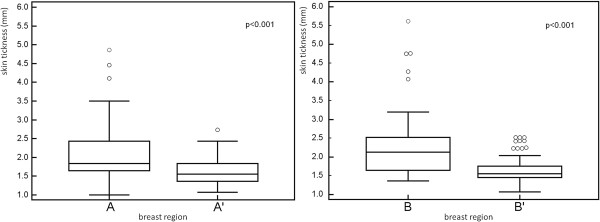
**Box and whisker plot for data comparison of measured skin thickness between treated and untreated breast; differences were statistically significant. A** corresponds to the irradiated breast, **B** corresponds to the boost region, **A’** and **B’** correspond to the mirror positions in the contra-lateral healthy breast.

**Figure 5:**
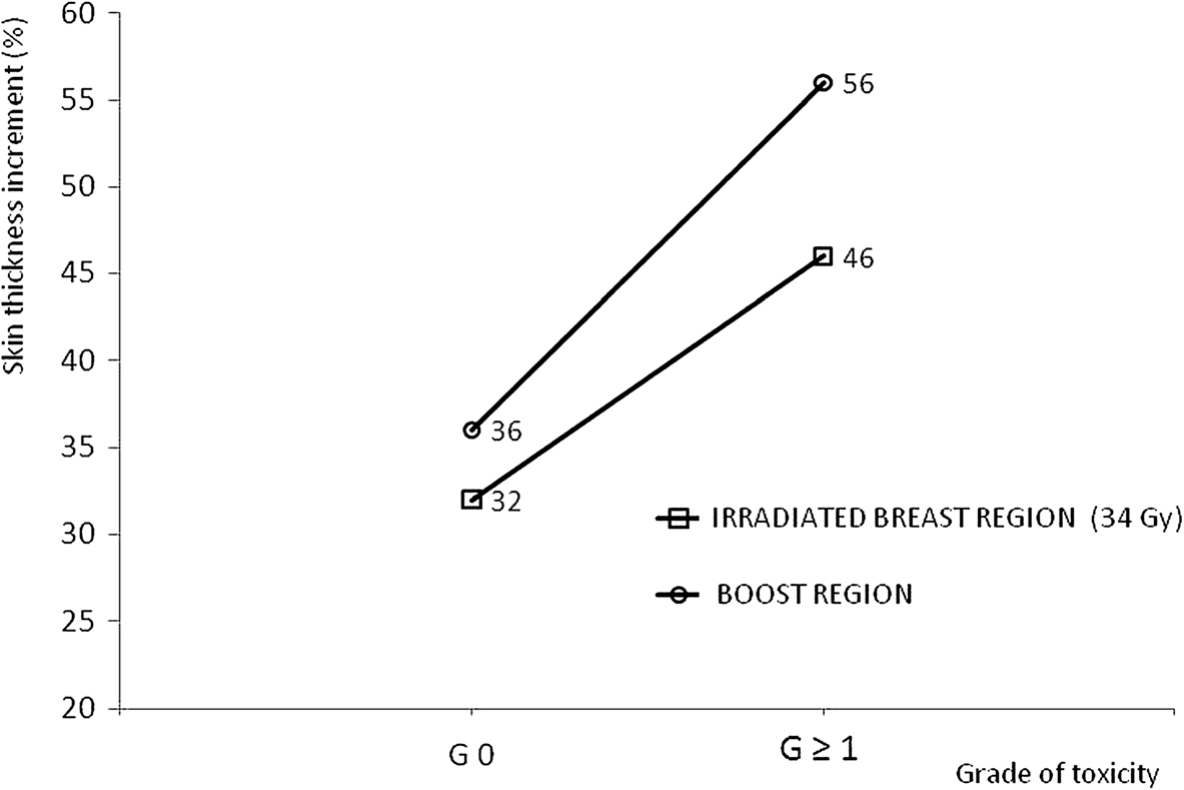
**Increment in skin thickness (%) in the boost (O) and in the irradiated breast (**□**) region (the 34 Gy region) for the different grades of toxicity.**

**Figure 6:**
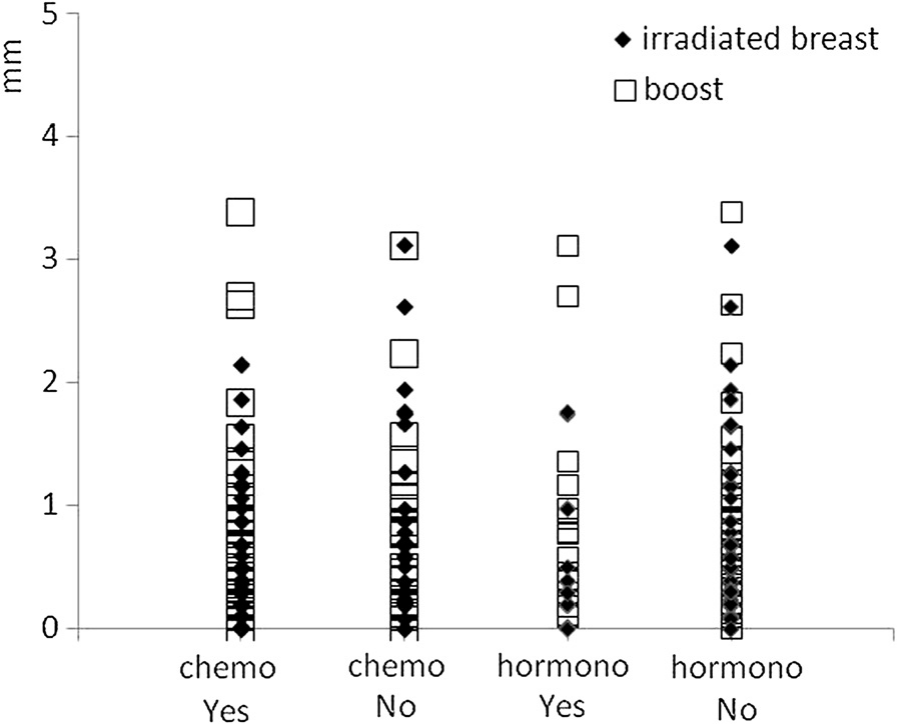
Scatter diagram of the correlation between previous adjuvant chemotherapy and/or concomitant hormonal therapy on skin thickenings.

All the patients were Caucasian. Patients’ median age was 62 years (range 31–79). Of the 89 patients included in the analysis, 37 had axillary nodes dissection and 52 had a sentinel lymph node biopsy. 36 patients (40%) received systemic chemotherapy, 68 (76%) hormonal therapy, and 23 (26%) patients received both. 8 (9%) patients received no adjuvant systemic therapy. Skin and subcutaneous toxicity scores as assessed at the same time of ultrasonographic examination are presented in Figure 3. A total of 13 patients (14.6%) developed at that time Grade ≥ 1 induration/fibrosis. No Grade 3 toxicity was observed. The time elapsed between the end of adjuvant radiotherapy and ultrasound examination ranged from 11.4 to 85.7 months (mean: 33.5, median: 20.5, standard deviation: 24.2). The measured mean skin thickness in the irradiated breast at 34 Gy (A) was 2.13 ± 0.72 mm while in the mirror region of the contra-lateral healthy breast (A’) was 1.61 ± 0.29 mm. The measured mean skin thickness in the irradiated boost region at 42 Gy (B) was 2.25 ± 0.79 mm versus 1.63 ± 0.33 mm in the corresponding region of contra-lateral healthy breast (B’). The mean increment in skin thickness respect to the counterpart in the healthy breast was 0.52 ± 0.67 mm and 0.62 ± 0.74 mm for the irradiated breast at 34 Gy and the boost region respectively. Differences in skin thickness measured in the boosted area (region B in Figure 2) and in the irradiated breast at 34 Gy (region A in Figure 2) were not significant. In Figure 4 data comparison for the measurements of skin thickness between treated and untreated breast are shown for both the irradiated breast and the boost region; differences in skin thickness were statistically significant (p < 0.001) for both examined regions. As expected the correlation between the increment in skin thickness in the boost region and the increment in skin thickness in the breast region resulted statistically significant (p = 0.0117). To assess the relevance of these data we investigated whether skin thickening as measured by ultrasonographic examination correlates with CTCv3 evaluation of radiation induced skin and subcutaneous tissue indurations/fibrosis. A significant direct correlation was found between the increment in skin thickness in the irradiated breast and in the boost region with fibrosis (G ≥ 1), with a p value of 0.0236 and 0.0164 respectively. In agreement with the correlation above reported we found that in the irradiated breast region the average increase in skin thickness was 32% among patients with Grade 0 fibrosis and 46% among patients with Grade ≥ 1 fibrosis. While in the boost region the average increase in skin thickness was 36% among patients with Grade 0 fibrosis and 56% among patients with Grade ≥ 1 fibrosis. The increment in skin thickness (%) in the boost and in the irradiated breast region for the different levels of toxicity is reported in Figure 5. Results of the evaluation of the role of previous adjuvant chemotherapy and/or concomitant hormonal therapy on skin thickening are shown in Figure 6. No significant correlation was found between skin thickening and systemic therapies, in particular for skin thickening in the treated breast at 34 Gy and in the boost region p was 0.340 and 0.411 for chemotherapy and 0.259 and 0.729 for hormonotherapy.

## Discussion

Several phase III randomized clinical trials [1-3] have evaluated the issue of hypofractionation in early-stage breast cancer showing that hypofractionated adjuvant whole breast radiotherapy after breast-conserving surgery offers equivalent results to those seen with normo-fractionated approach also representing an attractive treatment option because it allows for the shortened course of adjuvant RT. However concerns remain about the role of the boost dose in hypofractionated fashion on the overall treatment’s potential toxicity to such an extent that the ASTRO task force, who in 2011 developed an evidence-based guideline to provide direction for whole breast hypofractionation in clinical practice, did not reach unanimous consensus regarding a specific dose-fractionation scheme to use for the boost dose, therefore the ASTRO task force concluded that “on the basis of the published data and the collective expert opinion of the panel, boost doses of 10–16 Gy in 2-Gy fractions or 10 Gy in 2.5-Gy fractions were considered acceptable” [11]. On the other hand in the three randomized trials that contributed to clarify the role of hypofractionation in adjuvant whole breast radiotherapy the boost dose to the tumor bed was not prescribed [1] or was administered (at discretion of physician or according to local indications) in percentage ranging between 42% [2] and 60% [3] always at 2 Gy/fr to a total dose of 10 Gy in five fractions. In addiction the impact of boost dose on late toxicity was not separately analyzed. In our study 14% of patients developed ≥ G1 late toxicity, this result being in accordance with other published data [12]. Skin fibrosis is a common radiation-induced late effect usually scored by means of eye and palpation-based rating scales that are inevitably affected by examining physician subjective judgment with possible intra ed inter-obsever variability, the same is for cosmetic results or change in breast appearance judged using different, sometimes homemade, scoring systems. In fact the application of different toxicity scoring scales, in conjunction with the possibility of a subjective interpretation of clinical toxicity data, based on visual and tactile examinations, might explain discrepancies in toxicity results between different studies. H. Alexander et al. [13] in 1979 first reported on high frequency, high resolution ultrasonic echo technique as an accurate simple and noninvasive method for measuring full-thickness human skin. Recently T. Liu et al [14,15] have pointed out the role of high frequency ultrasound imaging as a reliable tool to assess late skin toxicity after breast radiotherapy also by change of skin thickness as a objective measure of the severity of fibrosis. Of note our study is the first one on the late skin toxicity assessment by quantitative ultrasonographic analysis after accelerated hypofractionated radiotherapy in women who underwent breast conserving surgery. Moreover in our cohort we analyzed whole breast as well as boost area radiation–induced late skin toxicity by quantitative ultrasonographic analysis through the correlation between skin thickness in the two “dose-levels” irradiated region (i.e., whole breast and boost area) and the mirror regions of the contralateral not irradiated healthy breast. In the paper by T. Liu et al [16] the ultrasonographic evaluation of radiation induced toxicity is reported in terms of skin thickness, Pearson coefficient and midband fit and the three parameters are said to be able to measure toxicity and correlate with the clinically RTOG scored one [17]. In our study only skin thickness was measured by ultrasonography and toxicity was scored with CTCv3 scale. Nevertheless our results are in agreement with the previous reported pilot study of breast cancer radiotherapy in which authors state that there is a “good correlation between skin thickness measurements and clinical assessment, suggesting this parameter’s ability to measure dermal injury”. Ultrasonographic examination was also used to try to clarify the role of boost dose with hypofractionated approach on late skin toxicity evaluating the burden of a single high boost-dose by means measurements of skin thickness in the boost region and in the non boost region of the irradiated breast. To the best of our knowledge none of study on high frequency ultrasound imaging as a consistent instrument to assess late radiotherapy skin toxicity have focused its attention on boost area. In our cohort there was no significant difference in skin thickness between boost (“42 Gy irradiated area”) and no boost region (“34 Gy irradiated area”) of the affected breast. So that it seems that the additional boost in a single high dose fraction does not contribute to enhance fibrosis detectable through an increase in skin thickness. This result could perhaps contribute to better define the feasibility of boost dose administration with hypofractionated approach. The authors recognize that a possible limitation of their study could be that the time between the end of radiotherapy and the ultrasonographic examination vary widely among patients but a minimum follow up of about 1 year was considered enough for late skin toxicity to be initially expressed. Another issue is whether the previous adjuvant chemotherapy, specially modern anthracycline-based and taxane-based regimes, as well as concomitant hormonal therapy might be responsible for worse fibrosis and adverse cosmesis. The effect of hypofractionation on cosmetic outcome and fibrosis in women who received this adjuvant systemic therapy was not separately assessed in the three prospective randomized trials mentioned above. T Hijal et al. [18] in a single-centre retrospective analysis reported that the rates of late skin toxicity were not significantly different in respect of adjuvant chemotherapy. In our cohort 38/89 patients received chemotherapy (mostly anthracycline-based and taxane-based regimes) before hypofractionated whole breast radiotherapy and no correlation was found between skin thickening and previous systemic therapies.

## Conclusion

Our study confirms that late toxicity evaluation by means of US is feasible, easy, not expensive and not highly time consuming and that is in agreement with clinical assed toxicity suggesting its widespread especially when patients are treated with new schedules of breast radiotherapy. In particular, as the use of hypofractionation increases and more and more frequently new schedules are tested in adjuvant WBI prospective trials, it could be crucial to have a quantitative easy reproducible tool for assessing and documenting late cutaneous reaction not affected by intra- and inter-observer variation in adjunct to physical examination based on eye and/or palpation. The results of the study in progress by Liu et al [14] on a breast cancer population “in which specific locations, such as the boost regions, will be separately examined” and the proposed investigation on hypofractionaction might confirm our conclusions. If this will be the case, giving a quantitative measure of toxicity and being possible to revaluate images, because stored and documented, this technique good play an important role in multicentric studies where using the same “language” should be encouraged.

## Abbreviations

WBRT: Whole breast radiotherapy; BCT: Breast conserving therapy.

## Competing interests

The authors hereby declare that they do not have any competing interest in this study.

## Authors’ contributions

PP and VL conceived and designed the study. CG, AMF, BS, MGP, VL, PP collected and assembled the data, AM performed the ultrasound examinations, VL performed the statistical analysis, VL and PP wrote the manuscript. LS gave support in the statistical analysis and in the final drafting of the paper. All authors read and approved the final manuscript.
